# Hashimoto’s thyroiditis and thyroid hormone sensitivity in euthyroid individuals: their association with carotid plaque in northeast China

**DOI:** 10.3389/fendo.2025.1605875

**Published:** 2025-09-17

**Authors:** Yanmeizhi Wu, Jingxue Sun, Yanan Ni, Minnan Wang, Hong Qiao

**Affiliations:** ^1^ Department of Endocrinology, The Second Affiliated Hospital of Harbin Medical University, Harbin, China; ^2^ National Health Commission (NHC) Key Laboratory of Etiology and Epidemiology, Harbin Medical University, Harbin, China

**Keywords:** carotid plaque, normal thyroid function, HT, thyroid hormone sensitivity, thyroid antibodies

## Abstract

**Background:**

Carotid plaque is a hallmark of systemic atherosclerosis, which underlies the pathology of cardiovascular and cerebrovascular events. Both thyroid hormones and Hashimoto’s thyroiditis (HT) affect carotid plaque formation, but their impact on individuals with normal thyroid function remains unclear.

**Methods:**

Clinical data, including demographics and medical history, were collected. Carotid and thyroid ultrasound, thyroid function tests, thyroid autoantibody measurements, and lipid profiles were performed. A retrospective analysis was conducted to explore the correlation of Hashimoto’s thyroiditis (HT) and thyroid hormone sensitivity with carotid plaque risk in euthyroid individuals, with additional subgroup analyses.

**Results:**

A total of 459 euthyroid patients were included. Thyroid hormone sensitivity indices were higher in the HT group than in the non-HT group. Significant differences in gender, age, smoking duration, diabetes mellitus (DM), coronary artery disease, ischemic stroke, and thyroid hormone sensitivity were observed between patients with and without carotid plaque. Among these factors, sex, age, coronary heart disease, and thyroid hormone sensitivity were identified as independent risk factors for carotid plaque. Differences in plaque size and thickness were noted between the HT and non-HT groups. Notably, in patients with DM, findings regarding plaque risk and characteristics diverged from those in other subgroups.

**Conclusion:**

In euthyroid individuals, HT impairs thyroid hormone sensitivity. Increased thyroid hormone sensitivity (as reflected by higher sensitivity indices) elevates carotid plaque risk. While HT may exert a modest protective effect against plaque formation, it contributes to the deterioration of plaque quality in patients with diabetes mellitus and dyslipidemia and elderly individuals with increased thyroid hormone sensitivity.

## 1 Introduction

Hashimoto’s thyroiditis (HT), also known as chronic lymphocytic thyroiditis, is a common chronic autoimmune thyroid disease characterized by autoimmune damage to thyroid tissue and significantly elevated levels of thyroid autoantibodies. The core antibodies include thyroid peroxidase antibody (TPOAb) and thyroglobulin antibody (TgAb). Currently, the prevalence of HT in the general population is approximately 5%, and it increases with age, peaking between 45 and 65 years (1). Notably, among HT patients, only 0.1%–2% present with clinically overt thyroid dysfunction, and 10%–15% have subclinical dysfunction (1); thus, most HT patients maintain normal thyroid function but are accompanied by significantly elevated titers of thyroid autoantibodies.

Cardiovascular disease (CVD) is a major global health threat, with significant regional disparities in its incidence. In China, Heilongjiang Province, in the northeastern region, a cold region with unique climatic and lifestyle characteristics, has a significantly higher incidence of cardiovascular disease than the national average (2). Atherosclerosis is the pathological basis of cardiovascular events such as coronary heart disease and ischemic stroke, and unstable intravascular plaques increase the risk of acute events (3). As an early marker of systemic atherosclerosis (4), carotid plaque holds important clinical significance, with approximately 25% of ischemic strokes caused by carotid stenosis (5). Epidemiological evidence indicates that HT is associated with carotid mesothelial thickening (6), and elevated levels of TPOAb are related to intracranial vascular remodeling (7), suggesting that HT patients face a higher cardiovascular risk. HT exerts multidimensional effects on the formation and progression of arterial plaques through two main pathways: on one hand, HT can cause abnormal thyroid hormone levels, which in turn affect plaque formation through metabolic disorders—thyroid hormones induce dyslipidemia, thereby promoting carotid intima thickening and plaque formation, and may further increase plaque risk by influencing blood pressure and glucose regulation; on the other hand, thyroid autoantibodies directly participate in plaque progression through immune regulation, inflammatory responses, and vascular remodeling (7). Therefore, focusing on the population in northeastern China to reveal region-specific insights into the relationship between HT and carotid plaque, enriching our understanding of the pathogenesis of cardiovascular disease in cold environments, holds important value for targeted prevention.

Traditional thyroid function indicators (e.g., free thyroxine, FT4) have limitations in assessing cardiovascular risk in euthyroid patients with HT—previous studies have reported conflicting findings (8–11). This has prompted researchers to propose thyroid hormone sensitivity indices, which can better reflect the body’s biological response to thyroid hormones. These indices include central sensitivity indices (T4RI, TSHI, TFQI) and peripheral sensitivity indices (T3/T4 ratio), with higher values indicating poorer sensitivity (12). Compared with traditional thyroid hormone levels, these sensitivity indices can more accurately capture subtle functional abnormalities of the thyroid axis in euthyroid individuals, thus being more promising in assessing CVD risk in HT patients. However, the role of thyroid hormone sensitivity in carotid plaque formation in euthyroid HT patients remains unclear.

Against this backdrop, this study retrospectively analyzed the clinical data of patients from the Department of Neurology at the Second Affiliated Hospital of Harbin Medical University, aiming to investigate the effects of HT and thyroid hormone sensitivity on the risk and nature of carotid plaque in euthyroid individuals. Further subgroup analyses were conducted in different populations to provide evidence in support of a deeper understanding of the role of thyroid status in the pathogenesis of carotid plaque.

## 2 Materials and methods

### 2.1 Collection of clinical case information

Clinical information was collected from patients with normal thyroid function who attended the Department of Neurology at the Second Hospital of Harbin Medical University, which included the following: 1) general information—ID, gender, age (years), weight (kg), blood pressure (mmHg), exposure and duration of smoking, and alcohol consumption (years); 2) past medical history—diabetes mellitus (DM), hypertension, coronary heart disease, and ischemic stroke; 3) carotid ultrasound—presence or absence of plaque and nature of plaque including size, thickness, and echogenicity; 4) thyroid ultrasound; 5) thyroid function and antibodies—free triiodothyronine (FT3), free thyroxine (FT4), thyroid-stimulating hormone (TSH), TPOAb, and TGAb; and 6) lipid levels—total cholesterol (TC), triglycerides (TG), high-density lipoprotein cholesterol (HDL-C), and low-density lipoprotein cholesterol (LDL-C). All examinations and test results based on this study were obtained from the Second Hospital of Harbin Medical University.

The inclusion criteria were as follows: i) normal thyroid function, i.e., FT3 (2.43–6.01 pmol/L), FT4 (9.01–19.50 pmol/L), and TSH (0.35–4.94 μIU/mL); (i) no iodine-containing medications or neck radiation exposure in the past 6 months; and iii) not pregnant, lactating, or experiencing growth disorders or psychiatric illnesses.

Assurance statements included 1) informed consent from all patients, 2) adherence to the ethical guidelines of the 1975 Declaration of Helsinki, and 3) prior approval from the institution’s ethics committee.

### 2.2 Diagnosis of HT and assessment of thyroid hormone sensitivity

The internationally accepted standard for HT diagnosis (13) primarily relies on elevated thyroid autoantibodies (particularly TPOAb > 300 IU/mL in our HT group vs. normal range in the non-HT group). While characteristic ultrasound findings (e.g., thyroid enlargement) support diagnosis, they are neither mandatory nor exclusionary per international guidelines. Our HT cohort included 1) typical cases with both antibody elevation and ultrasonographic features and 2) antibody-positive cases (TPOAb > 1,000 IU/mL) without ultrasound abnormalities. All equivocal cases were excluded.

Thyroid hormone sensitivity was assessed (12) through central indicators (T4RI = FT4 × TSH, TSHI = LN(TSH) + 0.1345 × FT4, TFQI = cdfFT4 − (1 − cdf TSH)) and peripheral sensitivity (T3/T4 ratio), with larger values indicating poorer sensitivity.

### 2.3 Evaluation of carotid plaque

Carotid plaque assessment via ultrasound includes evaluating the presence, size, thickness, and echogenicity (strong, isoechogenic, inhomogeneous, or hypoechogenic) of plaques, focusing on the largest plaque reported.

Furthermore, our study quantified carotid plaque burden using the carotid artery score (CAS), a comprehensive grading system based on ultrasound characteristics including plaque number (0 = 0, 1–2 = 1, ≥3 = 2 points), maximum thickness (<2mm = 0, 2–4 mm = 1, >4mm = 2 points), stability (hyperechoic/smooth = 0, hypoechoic/mixed = 1, ulcerated = 2 points), and stenosis degree (<50% = 0, 50%–69% = 1, ≥70% = 2 points). Total scores (0–8) were stratified as low (0–2), moderate (3–5), or high risk (6–8). Two blinded sonographers independently assessed plaques in all carotid segments using 7–15 MHz transducers, with discrepancies resolved by consensus.

### 2.4 Other variables

The diagnosis and grading of hypertension were based on the 1999 WHO classification criteria: grade 1 (systolic blood pressure 140–160 mmHg and/or diastolic blood pressure 90–100 mmHg), grade 2 (160–180 mmHg and/or 100–110 mmHg), and grade 3 (>180 mmHg and/or >110 mmHg). Dyslipidemia was diagnosed when any lipid levels were abnormal (TC ≥ 5.17 mmol/L, TG ≥ 1.7 mmol/L, HDL-C < 1.04 mmol/L, LDL-C ≥ 3.15 mmol/L). Individuals over 65 years were classified as elderly.

### 2.5 Statistical analysis

Raw data were checked for errors and analyzed using SPSS. Measurement data were presented as mean ± standard deviation, while count data were expressed as frequency (percentage). Statistical tests included *t*-tests, ANOVA with Tukey’s test for multiple comparisons, chi-square tests for percentages, logistic and linear regression for multifactor analysis, and Pearson correlation analysis. Corrected *p*-values were reported, with significance set at *p <*0.05.

## 3 Results

### 3.1 Clinical case review

A total of 459 patients were reviewed: 394 had carotid plaques and 65 had no detectable plaques. Of these, 119 had HT and 340 were non-HT. Detailed information is provided in **Table 1**.

**Table 1:** General characteristics of included cases.

Characteristics	Groups	Carotid plaque		*p* for single-factor analysis	*p* for multi-factor analysis
		Yes	No		
Sex				0.001	0.007
	Male	244(61.9%)	26(40.0%)		
	Female	150(38.1%)	39(60.0%)		
Age(years)				<0.001	<0.001
	≤65	170(43.1%)	46(70.7%)		
	>65	224(56.8%)	19(29.2%)		
Body weight(Kg)		68.99±14.87	70.35±13.42	0.510	0.417
Smoking				0.175	0.022
	No	303(76.9%)	55(84.6%)		
	Yes	90(22.8%)	10(15.3%)		
Duration of smoking(years)		7.97±16.14	2.66±9.14	0.012	0.009
Drinking				0.994	0.033
	No	331(84.0%)	55(84.6%)		
	Yes	60(15.2%)	10(15.3%)		
Duration of drinking(years)		4.81±13.04	3,28±10.35	0.381	0.012
Diabetes				0.006	0.066
	No	253(64.2%)	53(81.5%)		
	Yes	141(35.7%)	12(18.4%)		
Hypertension				0.165	0.549
	No	152(38.5%)	31(47.6%)		
	Yes	242(61.4%)	34(52.3%)		
Coronary heart disease				0.040	0.031
	No	308(78.1%)	58(89.2%)		
	Yes	86(21.8%)	7(10.7%)		
Ischemic stroke				0.010	0.158
	No	146(37.0%)	35(53.8%)		
	Yes	248(62.9%)	30(46.1%)		
Dyslipidemia				0.306	0.541
	No	102(25.8%)	13(20.0%)		
	Yes	291(73.8%)	52(80.0%)		
Hashimoto thyroiditis				0.061	0.619
	No	298(75.6%)	42(64.6%)		
	Yes	96(24.3)	23(35.3%)		
FT3		3.89±1.57	3.98±0.70	0.724	0.186
FT4		13.42±3.28	13.09±1.81	0.204	0.056
TSH		2.12±3.22	4.28±7.34	0.110	0.228
T4RI		26.89±33.80	45.76±53.94	0.031	0.241
TSHI		2.15±0.85	2.66±0.73	0.228	0.110
TFQI				0.116	0.003
	Q1	104(26.3%)	11(16.9%)		
	Q2	99(25.1%)	16(24.6%)		
	Q3	94(23.8%)	20(30.7%)		
	Q4	97(24.6%)	18(27.6%)		
T3/T4		0.29±0.05	0.31±0.08	0.008	0.155

A total of 459 patients were reviewed. Of these, 394 cases had carotid plaques and 65 cases without. Detailed information was shown in the table.

A total of 459 patients were reviewed. Of these, 394 cases had carotid plaques and 65 cases had no detectable plaques. Detailed information is shown in the table.

### 3.2 Thyroid hormone levels and sensitivity of HT

**Figure 1:**
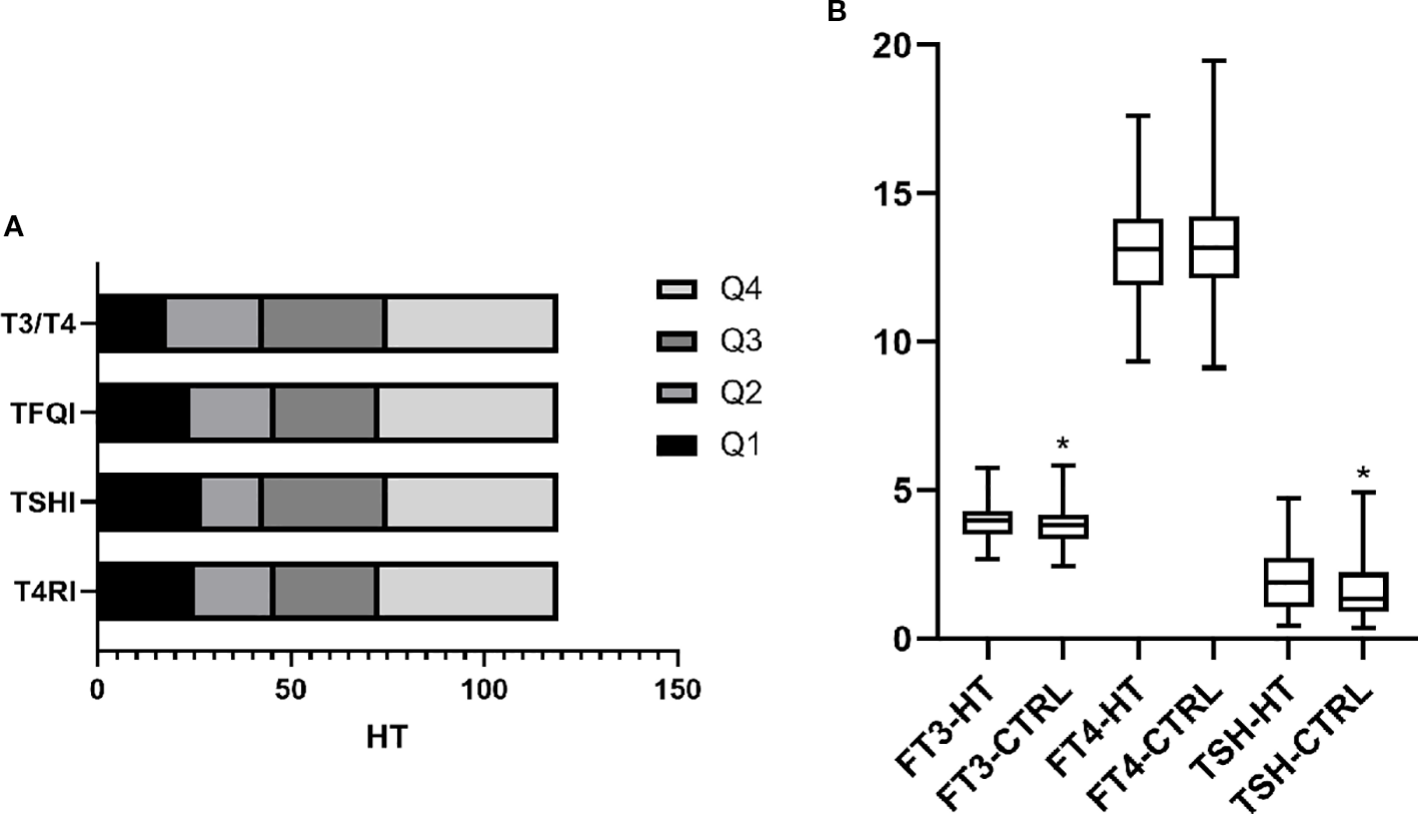
Levels and sensitivity of thyroid hormones in HT. **(A)** Indicators of thyroid hormone sensitivity were divided into four groups according to quartiles. The frequency of HT within each group is shown. The results show that HT in Q4 was significantly higher than in the other three groups. This indicates that even with normal thyroid function, HT has impaired thyroid hormone sensitivity, both centrally and peripherally. **(B)** Based on the diagnosis of HT, patients were divided into the HT and control (CTRL) groups. Comparing thyroid function between groups, FT3 and TSH were higher in the HT group than in the CTRL group, whereas no difference was found in FT4. *p <*0.05 was considered statistically different and marked with *.

Patients were divided into HT and non-HT groups to compare thyroid hormone levels and sensitivity indices. Results showed that T4RI, TSHI, TFQI, and T3/T4 were all higher in the HT group. Additionally, the HT group had elevated FT3 and TSH levels, with FT4 remaining unchanged. This indicates that HT patients, despite having normal thyroid function, exhibit impaired hormone sensitivity and higher thyroid hormone levels compared to non-HT patients. **Figure 1A** displays the frequency of HT across quartiles.

The correlation between thyroid hormone sensitivity and thyroid function/antibodies was analyzed. TSHI, TFQI, and T3/T4 correlated with FT3, FT4, and TSH, while T4RI correlated with FT3 and TSH but not FT4. T4RI and TSHI correlated with TPOAb, with TFQI and T3/T4 showing no association. Elevated T4RI aligned with hypothyroidism, while elevated TSHI and TFQI corresponded to hyperthyroidism. Notably, T3/T4 had a complex relationship, positively correlating with FT3 and negatively with FT4. These findings indicate the importance of T4RI and TSHI when considering thyroid antibodies in HT, as shown in **Supplementary Figure 1**.

### 3.3 Risk factors for carotid plaque

**Figure 2:**
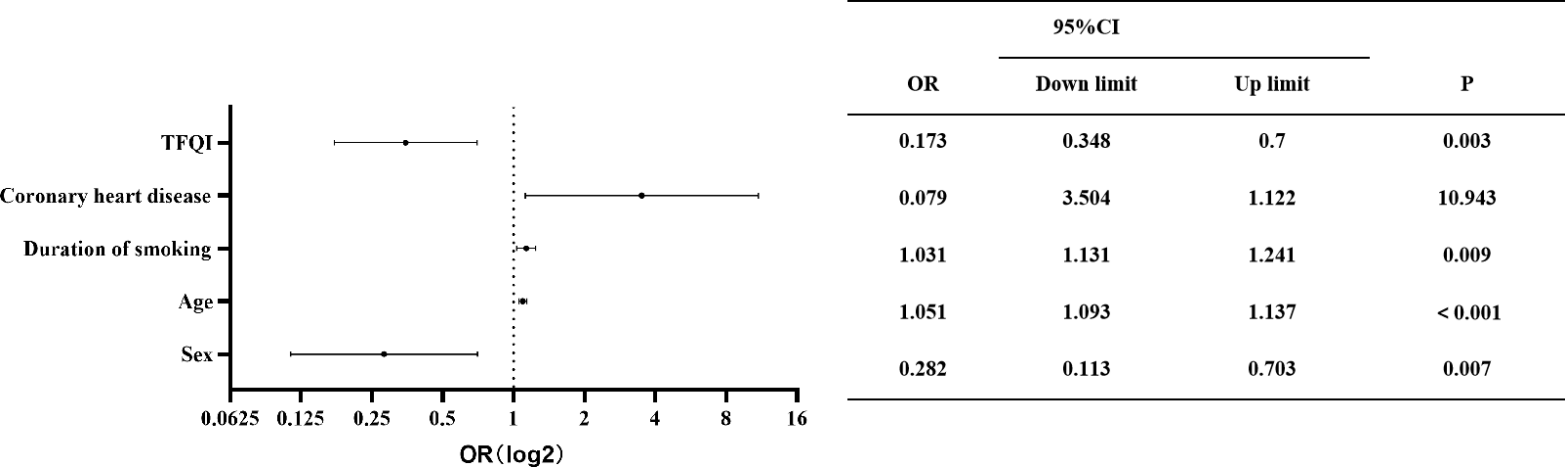
Independent factors of carotid plaque. OR, 95% CI, and *p*-value are shown for each independent factor. The horizontal coordinates are scaled in log2. Results showed that men and those with older age, higher central thyroid sensitivity, and previous coronary artery disease were at higher risk of carotid plaque.

Patients were categorized by the presence or absence of carotid plaque to compare risk factors. Univariate analysis identified significant factors, including sex, age, smoking duration, DM, coronary heart disease, ischemic stroke, T4RI, and T3/T4. Multifactorial analysis using logistic regression indicated that after accounting for interactions, independent risk factors for carotid plaque included sex, age, coronary artery disease, and TFQI. The odds ratio (OR) assessed relative risk, with factors having an OR greater than 1 as risk factors, while those less than 1 indicated protective effects. As shown in **Figure 2**, men, older individuals, and those with higher central thyroid sensitivity and a history of coronary artery disease are at increased risk for carotid plaque. Importantly, hypertension was not found to directly influence carotid plaque risk.

### 3.4 Effect of HT and thyroid hormone sensitivity on the nature of carotid plaque

Carotid plaques were identified in 394 individuals, with 96 HT patients and 298 without HT. Carotid ultrasound assessed plaque characteristics, including size, thickness, and echogenicity. Univariate analysis indicated differences in plaque size between the HT and non-HT groups. TFQI quartile grouping revealed size variations between groups, while T3/T4 levels differed between plaque thickness categories of <2mm and ≥2 mm. This suggests that individuals with carotid plaques and HT, alongside poorer central thyroid hormone sensitivity, exhibited larger plaques; those with HT and poorer peripheral sensitivity were more likely to have thicker plaques (≥2 mm).

**Figure 3:**
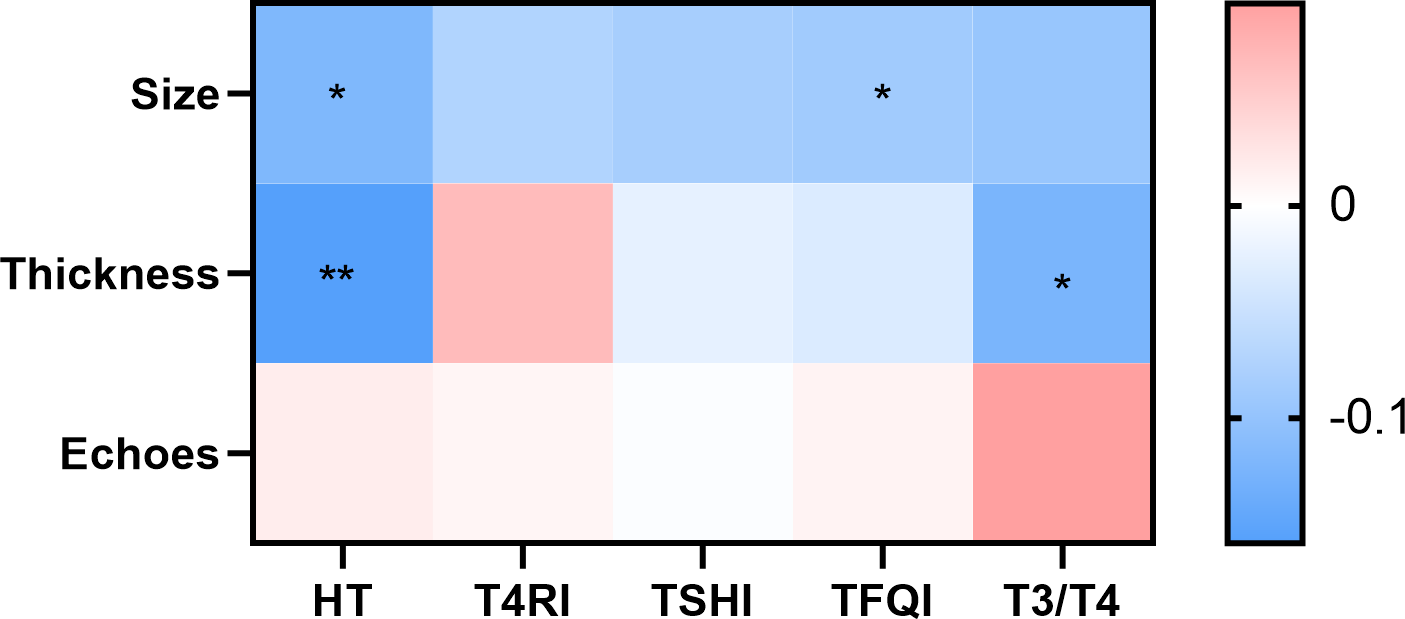
Contribution of HT and thyroid hormone sensitivity to carotid plaque properties. There was a difference in plaque size between the HT and non-HT groups. With TFQI quartile grouping, there were differences in plaque size between groups; T3/T4 and HT differed between the group with a plaque thickness <2 mm and the group with a plaque thickness greater than or equal to 2 mm. Color indicates correlation coefficient. *p <*0.05 indicates a statistical difference. *p < 0.05, **p < 0.01.

HT was correlated with smoking and alcohol consumption duration, indicating that these factors may contribute to HT development, where early cessation could help mitigate risks. Smoking exposure also negatively affected central thyroid hormone sensitivity. Multifactorial analysis confirmed that after controlling for confounding variables, T3/T4 remained associated with plaque size, while T4RI and TSHI were linked to plaque thickness. Thyroid hormone sensitivity served as an independent risk factor, with thicker plaques observed in individuals with reduced central sensitivity and larger plaques in those with reduced peripheral sensitivity. The results are shown in **Figure 3**.

### 3.5 Subgroup analysis

Subgroup analysis was performed on HT, DM, hypertension, dyslipidemia, coronary artery disease, ischemic stroke, and elderly populations to assess their effects on carotid plaques, as summarized in **Table 2**.

**Table 2:** Effect of HT and thyroid hormone sensitivity on carotid plaque.

Groups	Incidence of carotid plaque	Higher risk		Worse quality		Thyroid hormone sensitivity of HT
		HT	Sensibility	HT	Sensibility	
Total	85%		↑	+	↓	↓
Subgroups						
HT	80%		↑		↓	
DM	92%		↓		↑	↓
Hypertension	87%				↓	↓
Dyslipidemia	85%		↑		↑	↓
Coronary heart disease	92%				↓	↓
Ischemic stroke	89%				↓	↓
The elderly	92%			+	↑	↓

#### 3.5.1 HT

Of the 119 HT cases, 96 had carotid plaques. Univariate analysis indicated that T3/T4 correlated with plaque presence. Multifactorial analysis identified T4RI and TFQI as independent risk factors for plaque presence. Among the 96 HT patients with plaques, T4RI correlated positively with plaque thickness, while T3/T4 correlated with plaque echogenicity. This suggests that thyroid hormone sensitivity in HT patients affects both the occurrence and characteristics of carotid plaques, with thicker plaques associated with lower central sensitivity and poorer echogenicity linked to lower peripheral sensitivity.

#### 3.5.2 DM

Among 153 DM cases, 34 had concurrent hypertension, with 92% exhibiting carotid plaques (141 out of 153). The relationship between HT and thyroid hormone sensitivity was explored, revealing that T4RI, TSHI, TFQI, and T3/T4 levels were lower in the DM with HT group compared to those without HT. This indicates reduced central and peripheral thyroid hormone sensitivity in DM patients with HT. In the analysis of the diabetic population, the core focus was on the association between HbA1c and thyroid hormone sensitivity. When diabetic patients were stratified by a cutoff of 9% for HbA1c, significant differences in peripheral thyroid hormone sensitivity emerged. This suggests that in diabetic patients with concurrent HT, impaired peripheral thyroid hormone sensitivity may exacerbate poor glycemic control, forming a potential bidirectional association between thyroid function and metabolic regulation. Further analysis explored the relationship between HT, thyroid sensitivity, and the presence and characteristics of carotid plaques. Univariate analysis showed that HT, T4RI, TSHI, and T3/T4 ratio were all associated with the presence of plaques; however, initial multifactorial analysis identified age to be the only statistically significant factor. To exclude age-related confounding factors, we conducted a re-analysis after age-matching the diabetic population, and the results showed that T4RI, TSHI, and the T3/T4 ratio were all significantly correlated with the presence of plaques. Among the 141 diabetic patients with carotid plaques, central sensitivity indices exhibited a negative correlation with plaque size: the lower the thyroid hormone sensitivity, the larger the plaque volume. This indicates that impaired central thyroid sensitivity may drive plaque progression in the diabetic population. Notably, T4RI and TSHI were also associated with a history of alcohol consumption in this cohort. This suggests that alcohol intake may modulate central thyroid hormone sensitivity, thereby influencing glycemic control and carotid plaque formation in diabetic patients—an interaction that warrants further investigation to confirm.

#### 3.5.3 Hypertension

Among 276 hypertensive patients, 69 had combined HT, with 87% exhibiting carotid plaques (242/276). Those with hypertension and HT showed reduced thyroid hormone sensitivity. In this group, T3/T4 was positively correlated with plaque echogenicity; lower peripheral sensitivity correlated with reduced plaque echogenicity.

#### 3.5.4 Dyslipidemia

Among 343 dyslipidemia cases, 92 had combined HT, with 85% showing carotid plaques (291/343). Thyroid hormone sensitivity was impaired in this group. Univariate analysis indicated correlations between TSHI, T3/T4, and TFQI with plaque presence; however, multivariate analysis was not significant. Higher thyroid hormone sensitivity in dyslipidemia was associated with larger carotid plaques.

#### 3.5.5 Coronary heart disease

Among 93 coronary artery disease cases, 18 had combined HT, with 92% exhibiting carotid plaques (86/93). Differences in TSHI and T3/T4 were found between the HT and non-HT groups, while T4RI and TFQI showed no differences. In those with plaques, T4RI was positively correlated with plaque thickness; thicker plaques were associated with lower sensitivity.

#### 3.5.6 Ischemic stroke

Among 278 ischemic stroke cases, 73 had combined HT, with 89% showing carotid plaques (248/278). Thyroid hormone sensitivity was reduced in these patients. In those with plaques, TSHI negatively correlated with plaque size, while T4RI positively correlated with plaque thickness.

#### 3.5.7 The elderly

In a study of 243 elderly cases (>65 years), 62 had combined HT, with 92% showing carotid plaques (224/243). Thyroid hormone sensitivity was found to be worse in those with HT compared to non-HT individuals, and neither HT nor thyroid hormone sensitivity correlated with plaque presence. However, among those with carotid plaques, HT was positively correlated with plaque size and thickness, while TSHI showed a negative correlation with plaque size. Moreover, HT was associated with smoking and drinking history, indicating that tobacco and alcohol intake may increase the risk of developing HT, while early cessation might reduce this risk. Additionally, elevated TPOAb was linked to cerebral infarction risk, warranting caution in older adults. Among the 46 elderly individuals over 80 years, only one showed no plaque formation in the carotid arteries. Differences in central thyroid hormone sensitivity were noted between the HT and non-HT groups, with no differences in peripheral sensitivity. Univariate and multifactorial analyses identified TFQI as an independent risk factor for plaque presence. T3/T4 and HT were positively associated with plaque thickness, while TFQI was negatively correlated with age-related alcohol consumption, suggesting that timely abstinence from alcohol could help reduce TFQI levels and plaque risk.

## 4 Discussion

This study investigated the relationship between HT, thyroid hormone sensitivity, and carotid plaque in euthyroid individuals from northeast China, a cold region with a high burden of CVD. Our findings provide new insights into the role of thyroid status in carotid plaque pathogenesis, particularly regarding the mechanistic links between thyroid autoantibodies, hormone sensitivity, and atherosclerotic progression.

### 4.1 HT, thyroid autoantibodies, and carotid plaque development

Consistent with previous epidemiological evidence (6, 7), our results confirm that HT exerts multidimensional effects on carotid plaque formation and progression through dual pathways involving both thyroid hormone dysregulation and direct autoimmune mechanisms.

First, HT impairs thyroid hormone sensitivity, as evidenced by higher central (T4RI, TSHI, TFQI) and peripheral (T3/T4 ratio) sensitivity indices in the HT group compared to non-HT individuals. This impaired sensitivity aligns with subtle thyroid axis dysfunction, even in euthyroid states, and contributes to metabolic disturbances: reduced hormone sensitivity is associated with slower systemic metabolism, leading to dyslipidemia (characterized by cholesterol and triglyceride deposition in vascular walls) and subsequent carotid intima thickening. Our subgroup analyses further support that thyroid hormone sensitivity is an independent risk factor for carotid plaque, highlighting its role in linking HT to atherosclerotic risk beyond traditional thyroid function indices.

Second, thyroid autoantibodies—particularly TPOAb and TgAb—directly participate in plaque progression through immune-mediated mechanisms. TPOAb, as observed in our correlation analyses, activates Th1 cells and promotes the release of pro-inflammatory cytokines (e.g., IFN-γ, TNF-α) (7), accelerating atherosclerosis by inducing vascular inflammation (14). Additionally, TPOAb impairs endothelial function (consistent with its negative correlation with endothelial-mediated arterial dilation) and promotes vascular wall remodeling, weakening the vascular barrier and facilitating lipid deposition. TgAb, by interfering with thyroglobulin function, disrupts thyroid hormone synthesis and indirectly impairs lipid metabolism and vascular smooth muscle cell function, further contributing to plaque instability. Together, these findings reinforce that HT-associated autoimmunity is not merely a marker but an active driver of carotid plaque development in euthyroid individuals (15–17).

CAS, a comprehensive grading system integrating plaque number, maximum thickness, stability, and stenosis degree, provided critical insights into plaque nature in our study. We observed significant differences in plaque size and thickness between the HT and non-HT groups, with HT patients exhibiting larger and thicker plaques—features that align with higher CAS risk stratification (moderate to high risk). Notably, thyroid hormone sensitivity indices correlated with CAS components: poorer central sensitivity (higher TFQI) was associated with increased plaque size, while reduced peripheral sensitivity (lower T3/T4 ratio) was linked to greater plaque thickness. These findings highlight that CAS, combined with thyroid hormone sensitivity assessments, enhances the precision of CVD risk prediction in HT patients, enabling targeted interventions for those at the highest risk of acute events.

### 4.2 Subgroup-specific findings and clinical implications

Our subgroup analyses revealed distinct patterns in high-risk populations, with the DM subgroup showing the most notable nuances. In patients with DM, plaque risk and nature diverged markedly from other subgroups: while HT was associated with a potential reduction in plaque incidence, it strongly correlated with poorer plaque quality (larger size, increased thickness, and features of instability). This paradoxical pattern appears to be driven by interactions between HT-related thyroid dysfunction, impaired thyroid hormone sensitivity, and glycemic control—specifically, HbA1c levels. In our DM subgroup, higher HbA1c synergized with HT to exacerbate plaque pathology. Patients with HT and HbA1c ≥9% exhibited significantly higher thyroid hormone sensitivity indices and more severe plaque instability compared to those with HbA1c <9%, regardless of HT status (18). Mechanistically, poor glycemic control may amplify HT-associated immune-mediated vascular damage: elevated HbA1c induces oxidative stress and endothelial dysfunction, which synergize with TPOAb-mediated inflammation (7) to accelerate lipid deposition and vascular wall remodeling. Additionally, high HbA1c may further impair thyroid hormone sensitivity by disrupting hypothalamic–pituitary–thyroid axis regulation, creating a bidirectional loop that worsens both metabolic and vascular outcomes. Similarly, in elderly individuals and those with dyslipidemia, HT combined with increased hormone sensitivity exacerbated plaque instability, though without the same degree of glycemic interaction observed in DM patients. These results underscore that the impact of HT and thyroid sensitivity on carotid plaque is context-dependent, with glycemic control emerging as a critical modifier in DM cohorts. This highlights the need for tailored risk assessment in clinical practice—particularly in diabetic patients with HT, where integrating HbA1c monitoring with thyroid function and antibody testing may improve identification of those at highest risk of adverse vascular events.

### 4.3 Limitations and future directions

This study has several limitations. First, as a single-center retrospective analysis, selection bias may exist, and the findings may not fully generalize to other populations. Second, some confounding factors were not included, such as social status (which influences lifestyle factors like diet and healthcare access), medication history (e.g., lipid-lowering drugs, or anti-hypertensives), and hyperuricemia (but not assessed here) (12, 19, 20). Third, the cross-sectional design prevents causal inference, and longitudinal studies are needed to clarify the temporal relationship between HT, hormone sensitivity, and plaque progression (21). Future studies should focus on addressing the aforementioned limitations and further explore interventional strategies or approaches to optimize thyroid hormone sensitivity at the mechanistic level, which may provide novel therapeutic avenues for reducing carotid plaque risk in HT patients.

## 5 Conclusion

In euthyroid individuals, HT impairs thyroid hormone sensitivity and drives carotid plaque development through autoimmune and metabolic pathways. The CAS score, combined with thyroid sensitivity assessments, enhances risk stratification, particularly in high-risk subgroups like DM patients and the elderly. Addressing unmeasured confounders in future studies will further refine our understanding of this relationship, ultimately improving CVD prevention in HT populations.

## Data Availability

The original contributions presented in the study are included in the article/**Supplementary Material**. Further inquiries can be directed to the corresponding author.
