# Supplementary material for: Hashimoto’s thyroiditis and thyroid hormone sensitivity in euthyroid individuals: their association with carotid plaque in northeast China
Source: Front Endocrinol (Lausanne). 2025 Sep 17;16:1605875. doi: 10.3389/fendo.2025.1605875 (PMC12483847; doi:10.3389/fendo.2025.1605875)
Supplement: Supplementary file 1 [file DataSheet1.docx]

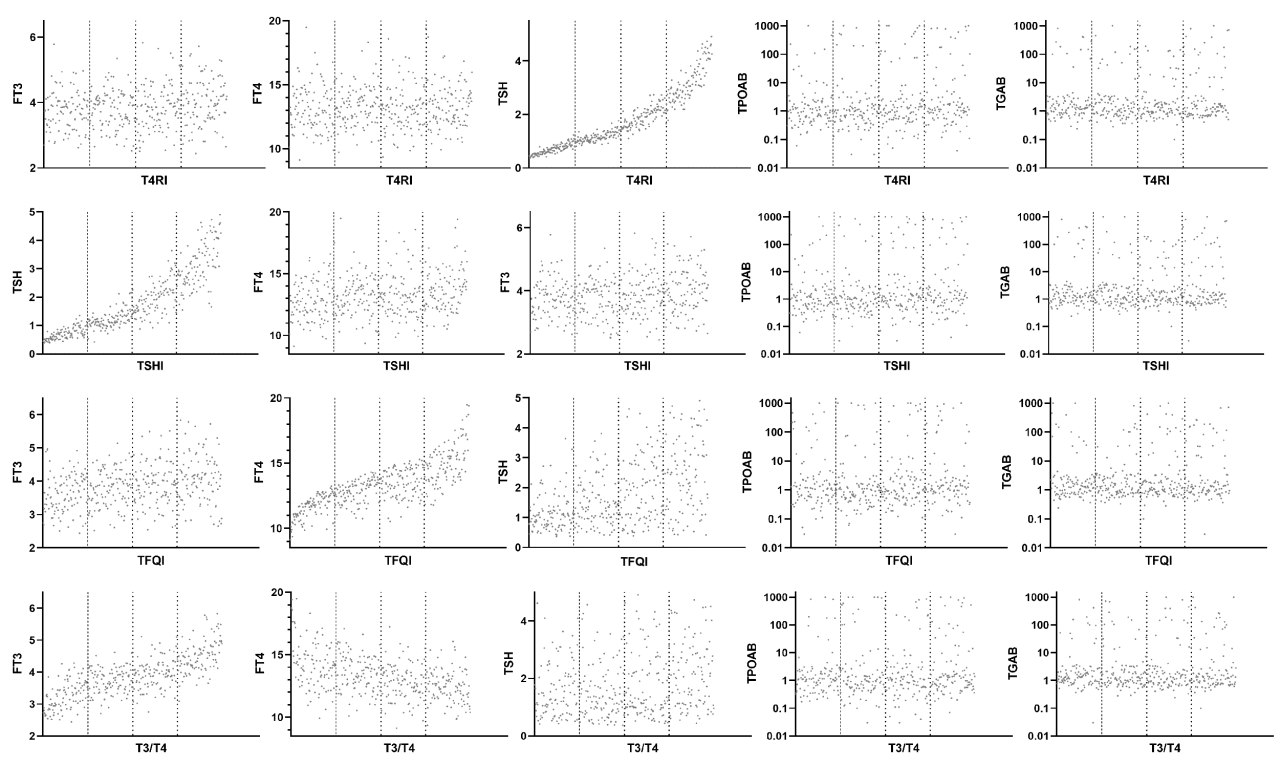


**Supplementary figure 1 Correlation of thyroid hormone sensitivity and thyroid status.**

It was found that TSHI, TFQI, and T3/T4 correlated with FT3, FT4, and TSH, while T4RI correlated with FT3 and TSH, but not with FT4. Also, T4RI correlated with TPOAb and TGAb, while TSHI correlated with TPOAb. TFQI and T3/T4 were not associated with the two antibodies.
